# Introduction of advanced laparoscopy for peritoneal dialysis catheter placement and the outcome in a University Hospital

**DOI:** 10.1007/s11255-021-03020-4

**Published:** 2021-10-18

**Authors:** J. H. H. van Laanen, E. J. Litjens, M. Snoeijs, M. M. van Loon, A. G. Peppelenbosch

**Affiliations:** 1 Department of Vascular Surgery, Maastricht University Medical Center, Maastricht, The Netherlands; 2 Department of Internal Medicine, Division of Nephrology, Maastricht University Medical Center, Maastricht, The Netherlands; 3 Department of Operative Care and Vascular Surgery, Maastricht University Medical Center, P.O. Box 5800, 6202 AZ Maastricht, The Netherlands

**Keywords:** Peritoneal dialysis, Surgical technique, Advanced laparoscopy, Functional outcome, Omentopexy, Catheter fixation

## Abstract

**Background:**

Peritoneal dialysis (PD) catheters can be obstructed by omental wrapping or migration, leading to catheter malfunction. Multiple catheter placement techniques have been described. Advanced laparoscopy with fixation of the catheter and omentum has been reported to improve functional outcome compared to basic laparoscopy without fixation. This feasibility study describes surgical technique, complications, and comparison of the functional outcome of advanced versus basic laparoscopic catheter placement.

**Methods:**

Between July 2016 and April 2019, the advanced laparoscopy technique was applied in all eligible patients. Two experienced surgeons placed the catheters in a standardized procedure. Peri-operative complications and functional outcome of the catheter were scored. Results were compared to a historical cohort retrieved from our RCT performed earlier using basic laparoscopy.

**Findings:**

The basic laparoscopic group (BLG) consisted of 46 patients and the advanced laparoscopic group (ALG) of 32. Complication rate in both groups was similar and low with 7% in the BLG and 6% in the ALG (*p* = 1.0). There was a trend toward better functional catheter outcome in the ALG (88%) compared to the BLG (70%) (*p* = 0.1). Part of the catheter failures in the ALG could be related to the learning curve. After revision surgery, 94% of patients in the ALG had a functional catheter. These findings lead to the set-up of a multi-center randomized-controlled trial, currently running, comparing basic to advanced laparoscopic techniques.

## Introduction

Peritoneal dialysis (PD) requires insertion of a peritoneal dialysis catheter into the abdominal cavity. Functional outcome can be defined as the uncomplicated inflow and outflow of dialysate, and is the primary outcome measure for a PD catheter. Functional outcome can be endangered by complications during or after catheter placement. Post-operative complications can be obstruction of flow through the catheter, catheter migration, fluid leaks, erosion of catheter into viscera, and sclerosing or bacterial peritonitis [1]. Various causes for catheter obstruction are identified such as omental wrapping, adhesions, and catheter migration [2, 3].

Several surgical techniques including open, blind percutaneous, peritoneoscopic, and laparoscopic PD catheter placement have been described [4–6]. These techniques have been developed over the years to decrease complications.

Laparoscopy compared to open procedure has several advantages that are associated with improvement of functional outcome by reducing catheter-related complications. An advantage of laparoscopy includes direct visualization during surgery. In addition, during advanced laparoscopic surgery, additional procedures as adhesiolysis, catheter fixation, and omentopexy can be performed [7, 8].

Fixation of the omentum to the abdominal wall of the upper abdomen (omentopexy) during laparoscopic catheter placement might prevent omental wrapping, thereby preventing catheter dysfunction. Omentopexy was described by Ögünc et al. and several studies about omentopexy and prevention of catheter dysfunction have been performed [9–11]. To prevent catheter migration, favorable outcomes after catheter fixation to the lower abdominal wall have been described [12, 13].

The randomized-controlled trial we conducted in our center in 2010–2016 demonstrated equal clinical success rates between open and laparoscopic catheter placement. However, no advanced laparoscopic techniques were applied in this trial [6]. Because of the disappointing results of laparoscopy and the reported advantages of advanced laparoscopic techniques in catheter placement, we decided to conduct a feasibility study in our center adding catheter fixation to the abdominal wall and omentopexy to our standard laparoscopic procedure including rectus sheath tunneling. Surgical technique, complications, and comparison of the mechanical outcome of the new techniques versus basic laparoscopic placement are described.

## Materials and methods

### Patient selection

We included all consecutive patients with end-stage renal disease who were eligible for a peritoneal dialysis (PD) catheter after finishing our randomized-controlled trial (RCT) in March 2016 [6]. Patients with a life expectancy of less than 1 year and patients in need for abdominal cavity surgery not related to catheter insertion were excluded. Patient history was taken and physical examination was performed. Previous abdominal surgery was not considered an exclusion criterion. Abdominal wall or incisional hernia was corrected with a mesh during PD catheter placement. Patients were informed about our previous RCT and its outcome and the presumed benefit of fixating the catheter and the greater omentum. The possible complications were explained, as well. After informed consent was obtained, patients were referred to the anesthesiologist for further screening before they were scheduled for surgery. Patients could participate only once in the study.

### Surgical procedure

All patients were operated on by one or both of two surgeons (AP and JvL). Both are experienced laparoscopic PD surgeons and have performed over 65 laparoscopic PD catheter placements before introducing the advanced laparoscopic techniques. All patients had the desired exit site marked by the PD nurse pre-operatively. The PD catheter was always a two cuff coiled-tip catheter. The exit site always faced downwards. Adhesiolysis was only performed if adhesions prevented the planned route of the catheter. All catheters were tested at the end of the procedure by installation of 1.25 L of Icodextrin 4% and aspiration of 200 ml hereafter. The rest of the solution was left in place to prevent adhesions [2].

If an abdominal wall hernia was present, it was laparoscopically corrected with a composite mesh with > 3 cm overlap. Hereafter, the catheter was placed in the preferred position in the lower abdomen.

In the first patients, only one of both fixation techniques was used. Later on, with more experience, both procedures were performed in one operation. The catheter was fixated with a non-absorbable suture which was usually a Prolene suture. The omental fixation was performed with non-absorbable (Prolene or Mersilene) or absorbable sutures (Vicryl). The latter was thought to be sufficient because of the sterile inflammation process that will take place and will create a tight adhesion from the omentum to the abdominal wall. In case the omentum was so small that it could not reach the desired position for fixation or could not reach the position of the catheter in the lower abdomen, it was not fixated and left in place. Also, if the omentum was already fixated by adhesions from previous surgery, it was untouched. We did not consider epiploic appendectomy or colopexy.

**Fig. 1:**
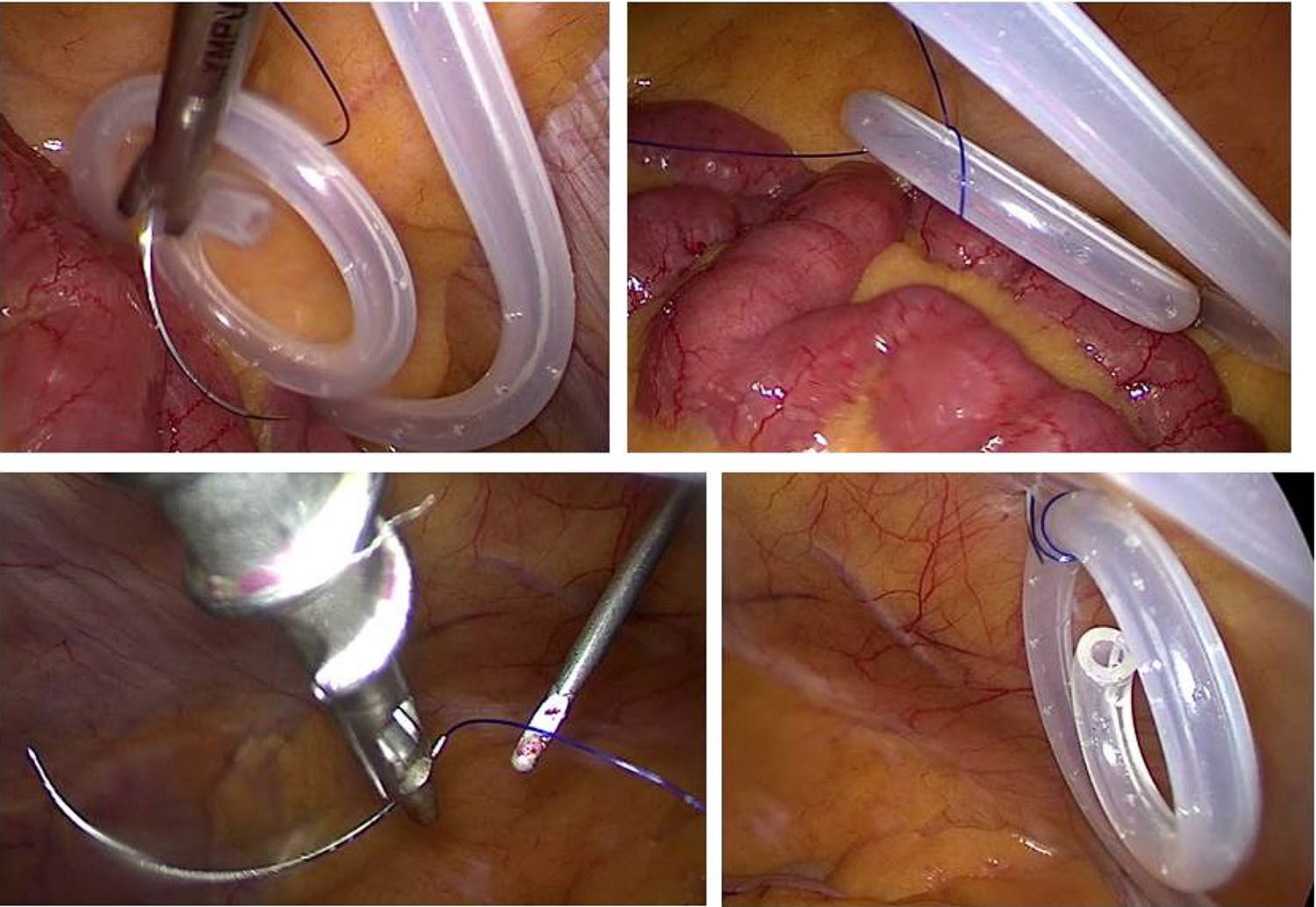
Catheter fixation. Upper left: needle wrapped around the catheter with needle holder. Upper Right: suture positioned around the catheter. Lower left: suture grasped with endoclose. Lower Right: catheter fixation finalized

**Fig. 2:**
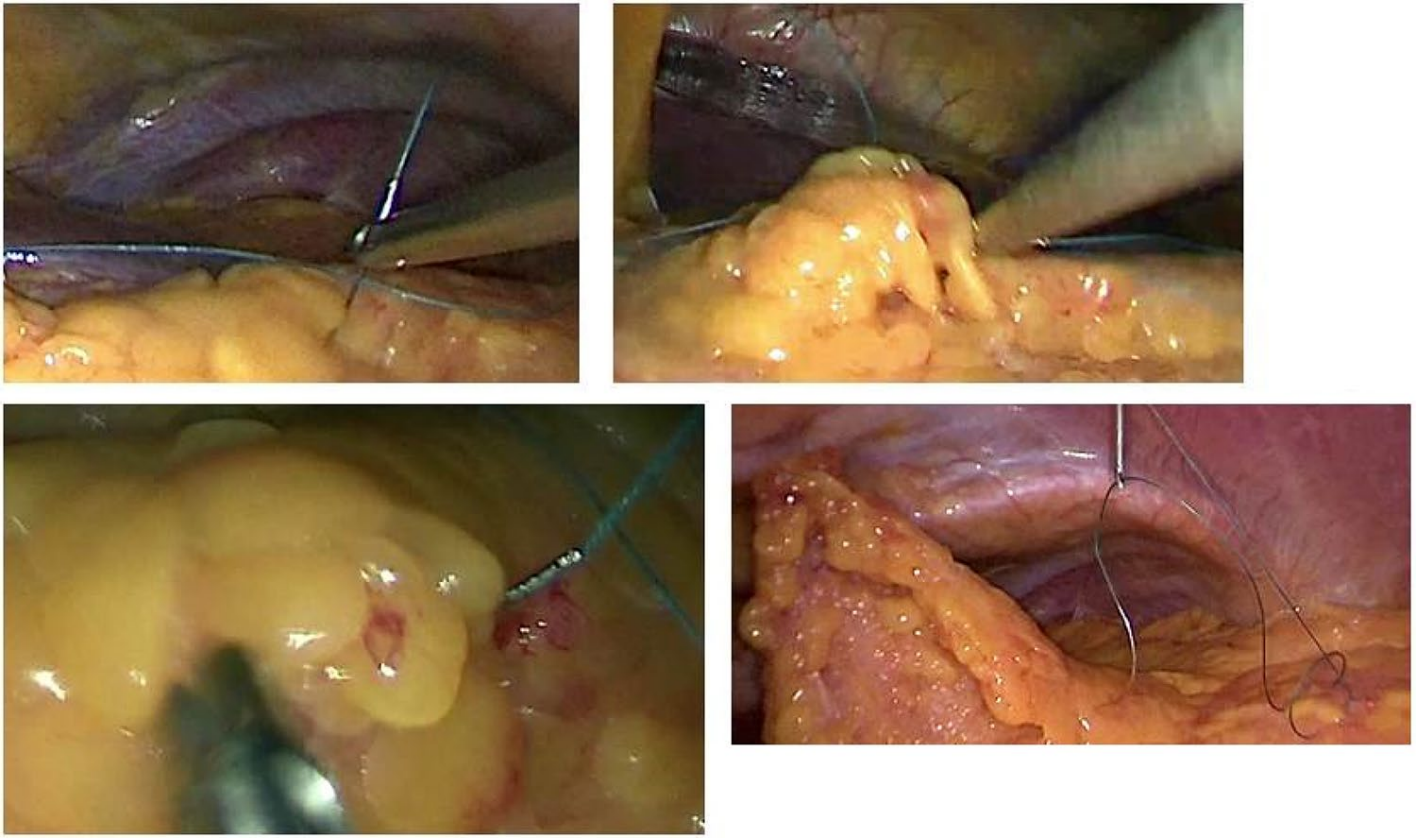
Omentopexy. Upper left: needle introduced into the abdomen and grasped with needle holder. Upper Right: needle through the omentum. Lower left: needle retrieved with needle holder after placed through the omentum. Lower Right: one fixation finalized; other suture retrieved with endoclose

Prophylactic antibiotic, one gram of cephalosporins, was administered pre-operatively. After general anesthesia and sterile exposure of the abdomen, the desired position of the subcutaneous track and cuffs were marked on the abdominal wall. Then, a 10 mm Hason trocar was introduced in the right hemi-abdomen under direct vision. A pneumo-peritoneum of 12–14 mmHg was created. Now, a 10 mm 30 degree angle laparoscope was introduced for inspection. Hereafter, a 5 mm working trocar was introduced in the right hemi-abdomen for introducing graspers and needle holders. If a hernia was present, the 5 mm working trocar was exchanged for a 12 mm working trocar to introduce a composite mesh with two-to-four pre-placed sutures for proper placement. Fixation of the mesh was with absorbable tackers after appropriate placement with an endoclose of the pre-placed sutures. A 7 mm trocar was introduced at the desired position of the subcutaneous catheter curve. The trocar tip was placed at the transversalis fascia under vision, then tunneled through the rectus sheath for 4–6 cm, and finally introduced into the abdominal cavity at the position where the deep cuff should be placed. Patients were placed in the Trendelenburg position before the catheter was introduced through the 7 mm trocar using a stylet. Under direct vision, the catheter was placed at the desired position in the abdominal cavity. The deep cuff was introduced into the abdominal cavity. The 7 mm trocar was then removed and the deep cuff of the catheter was retrieved into the preperitoneal space. The proximal cuff was placed in the subcutaneous layer more than 2 cm from the exit site, which was usually also at the left side of the abdominal wall. The catheter was fixated to the anterior abdominal wall in the midline with non-absorbable sutures using the endoclose (Fig. 1). One side of a prolene thread was introduced into the abdominal cavity with an endoclose device through a stab skin incision just above the upper margin of the urinary bladder. The thread was looped around the curved catheter tip twice with a grasper. The endoclose was then re-introduced into the peritoneal cavity through the same skin incision, but a second abdominal wall puncture and the thread were retracted. Then, both ends of the thread were knotted with 1–2 mm free space between catheter and abdominal wall and the knot rests in the subcutaneous tissue on the rectus fascia. The omentum was fixated to the anterior abdominal wall in the epigastric area (Fig. 2). For the omental fixation, a curved needle of a mersilene thread was manually straightened outside the body and the needle was introduced through the abdominal wall with a needle holder. The needle was grasped by a needle holder. An additional 5 mm working trocar was introduced for a grasper to lift the omentum, so a suture could be made. After fixation, the needle and thread were removed by an endoclose. In some cases, the endoclose was directly pushed through the omentum without the use of a needle. If necessary, an extra 5 mm working trocar was introduced for holding and grasping the omentum.

### Outcome measures

After the procedure, all patients had an abdominal X-ray to conform the position of the PD catheter. There were no re-interventions planned even if the position was not optimal (in the lower abdomen). Two weeks after insertion, the catheter was tested in the outpatient clinic and training was started. As per protocol, catheters were tested with low volumes of dialysate (250 ml). In case of a concurrent hernia repair, testing and training were started after 4 weeks. We evaluated the mechanical outcome of the catheter when first used. Technical success was defined as unobstructed inflow and outflow of dialysate without the need for revision surgery. Dialysate leakage from wounds, infections of the tunnel track, exit site, or the catheter itself during hospital stay and at outpatient clinic follow-up were scored.

Total catheter survival in time was scored. Catheter survival was scored as all mechanical functional catheters without the need for removal due to peritonitis, abdominal surgery, or inadequate peritoneal dialysis. Patients were censored for death (with mechanical functioning PD catheter), kidney transplant, and for patient preferences (switch to hemodialysis with functional PD catheter).

Patient demographics and mechanical outcome of the catheter of this study group were compared with data and outcome in our historical cohort of patients included in the randomized -controlled trial that compared open to basic laparoscopic PD catheter placement with rectus sheath tunneling. Patients from the historical RCT will be called the basic laparoscopic group (BLG) and those from the study group will be called the advanced laparoscopic group (ALG).

### Statistical analysis

Continuous variables are expressed as means with standard deviation (SD). Differences were calculated using the Mann–Whitney *U* test or Fisher’s exact test when appropriate. Survival analysis was performed using the Kaplan–Meier method. Two-sided testing was performed and a *p* value < 0.05 was considered statistically significant. Statistical analysis was performed using SPSS version 24 (IBM Corporation, Armonk, NY, USA).

## Results

From July 2016 up to October 2019, we performed advanced laparoscopy for PD catheter placement. In the early experience, only catheter fixation or omental fixation was used, and after six cases, with more experience, we started to use both techniques in one procedure. In this period, we treated 32 patients in the ALG. In Table 1, the patient characteristics are presented and compared to the BLG data. There were no significant differences between both groups. In Table 2, the operative and post-operative characteristics are depicted. There is an expected statistically significant difference for operation time in favor of the BLG. Fixating the omentum and catheter takes an extra 30 min (mean operating time ± SD in minutes for the BLG was 38.3 ± 15.3 compared to 69.2 ± 26.9 for the ALG; *p* < 0.001). The number of hernia repairs and adhesiolyses was similar in both groups. The mean hospital stay in both groups was approximately 3 days; however, most patients stayed 1 day or less in both groups (28 patients in the BLG and 20 patients ALG). The number of post-operative complications is low with less than 10% in both groups and mostly minor complications. There were no deaths in both groups.

**Table 1:** Patients’ characteristics

		Advanced laparoscopic group	Standard laparoscopic group	*p* value
Patients	*N*	32	46	
Male	*N* (%)	18 (56%)	29 (63%)	0.64
Age (years)	Mean ± SD	60.8 ± 13.7	62.6 ± 14.1	0.60
Hypertension	*N* (%)	25 (78%)	35 (76%)	1.0
Diabetes	*N* (%)	12 (38%)	13 (28%)	0.46
Heart failure (EF < 40%)	*N* (%)	4 (13%)	6 (13%)	1.0
Creatinine (umol/L)	Mean ± SD	556 ± 171	551 ± 254	0.46
Body mass index (kg/m^2^)	Mean ± SD	25.6 ± 4.3	26.5 ± 5.1	0.47
Previous abdominal surgery	*N* (%)	21 (66%)	22 (48%)	0.17
Previous median laparotomy	*N* (%)	10 (31%)	16 (35%)	0.81
Hemodialysis	*N* (%)	12 (38%)	12 (26%)	0.32
Previous PD catheter	*N* (%)	8 (25%)	8 (17%)	0.57

**Table 2:** Operative characteristics and complications

		Advanced laparoscopic group	Standard laparoscopic group	*p* value
Patients	*N*	32	46	
Operation time (minutes)	Mean ± SD	69.2 ± 26.9	38.3 ± 15.3	< 0.001*
Procedure performed				
Adhesiolysis	*N* (%)	7 (22%)	4	0.15
Hernia repair	*N* (%)	6 (19%)	2	0.18
Only fixation catheter	*N* (%)	7^a^	–	
Only fixation omentum	*N* (%)	5	–	
Fixation catheter and omentum	*N* (%)	20	–	
Hospital stay (days)	Mean ± SD	3.1 ± 6.5	3.2 ± 7.5	0.18
Mortality	*N* (%)	0	0	
Morbidity	*N* (%)	2 (6%)	3 (7%)	1.0
Bleeding	*N*	–	1	
Cardiac event (non-fatal)	*N*	–	1	
Wound leakage	*N*	–	1	
Trocar hernia	*N*	1	–	
Exit site infection < 2 weeks	*N*	1	–	

*Statistically significant difference

^a^In one patient, the omentum was already fixated to the abdominal wall with adhesions; in one patient, the omentum was considered too small to reach the catheter

**Table 3:** Mechanical outcome after PD catheter implantation

		Advanced laparoscopic group	Basic laparoscopic group	*p* value
Patients	*N*	32	46	
Mechanical functioning catheter	*N* (%)	28 (88%)	32 (70%)	0.1
Virgin Abdomen	*N* (%)	11	24	
Mechanical functioning catheter	*N* (%)	10 (91%)	15 (63%)	0.12
Previous abdominal operation	*N* (%)	21	22	
Mechanical functioning catheter	*N* (%)	18 (86%)	17 (77%)	0.70
Previous median laparotomy	*N* (%)	10	16	
Mechanical functioning catheter	*N* (%)	8 (80%)	11 (69%)	0.67
Previous implantation of PD catheter	*N* (%)	8	8	
Mechanical functioning catheter	*N* (%)	7 (88%)	7 (88%)	1.0

Regarding the primary outcome of mechanical function of the catheters, there was no statistically significant difference between both groups with a technical success rate of 70% (32 patients) in the BLG compared to 88% (28 patients) in the ALG (*p* = 0.1) (Table 3). If we focus on the reasons for failure in the ALG (Table 4), two catheters failed because of a technical problem that was resolved during re-operation. In one of these patients, the subcutaneous tunneled part caused an obstruction due to kinking not observed in supine position during placement. In the other patient, the knot in the suture for fixating the catheter failed and the catheter migrated to the upper abdomen. Both patients were re-operated, the curve was corrected in one, and the catheter was fixated in the other and both patients had a mechanical good functioning catheter afterward.

**Table 4:** Reasons for failure of the PD catheter

		Advanced laparoscopic group	Basic laparoscopic group
Patients	*N*	4	14
Omental wrapping/bowel entrapment	*N*	1	7
Migration of catheter	*N*	1	3
Bleeding and removal of catheter	*N*		1
Dialysate leakage	*N*		1
Bend of catheter too steep causing obstruction	*N*	1	
Peritonitis causing removal of catheter	*N*		1
Adhesions	*N*	1	1

The other two catheter failures can be attributed to failure of the advanced techniques to prevent catheter obstruction. In one patient, only omental fixation was performed during primary surgery. After revision surgery with fixation of the catheter, it still malfunctioned because of small bowel wrapping around the catheter as seen with a diagnostic laparoscopy in a third procedure. This patient switched to hemodialysis. In the other patient, the advanced technique was well conducted, but the curled portion of the catheter was covered by the peritoneum of the abdominal wall. We corrected the position of the suture to fixate the catheter to a more cranial site at the straight segment of the catheter, and hereafter, the catheter had a good mechanical function.

In our former publication, the BLG follow-up is described in detail [6]. To summarize these findings, of 14 technical failures, 8 patients had a re-operation and 6 of these led to technical success.

**Fig. 3:**
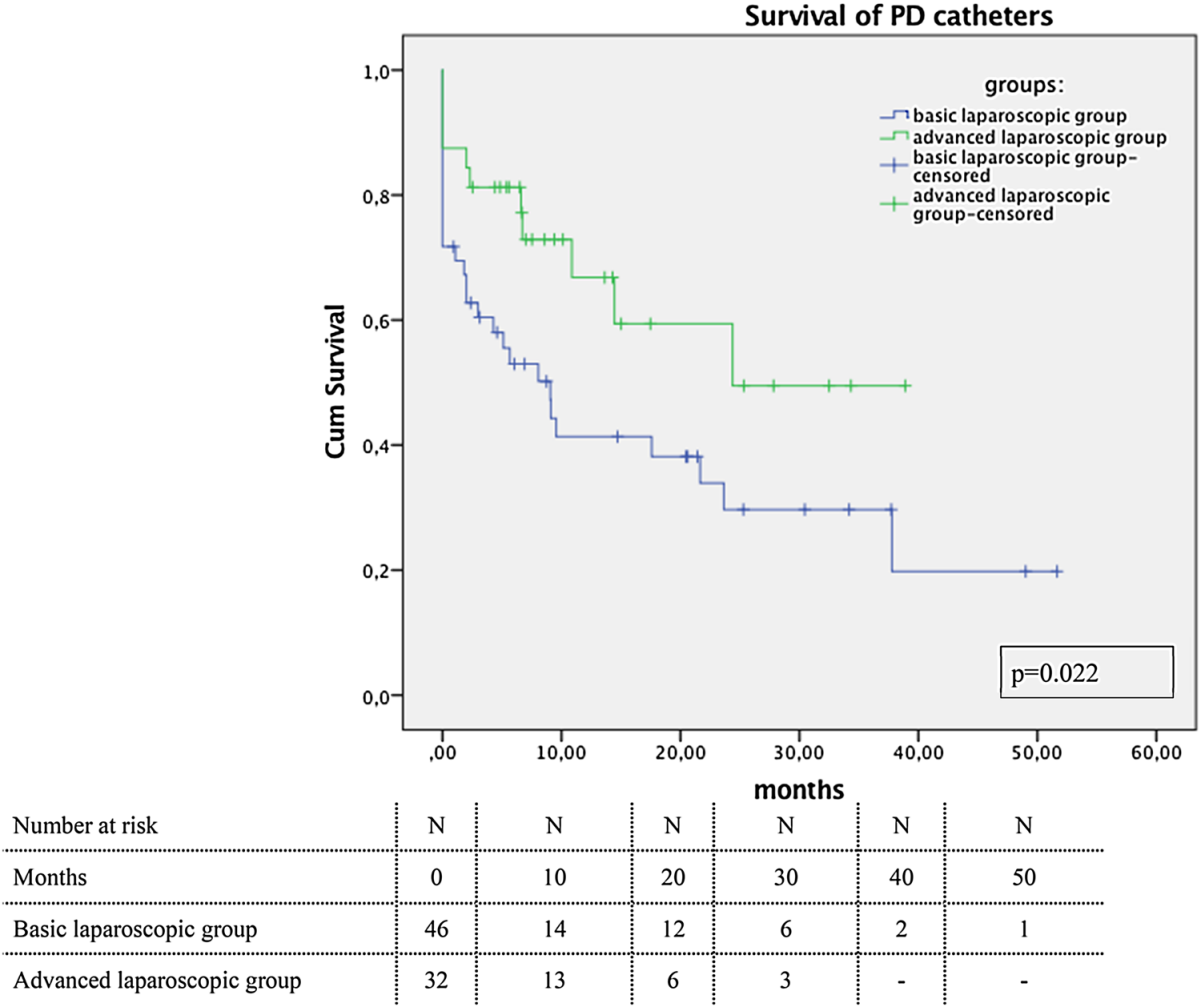
Survival plot of the PD catheters for both groups

Figure 3 demonstrates the survival curve in time of both groups for the PD catheter. As demonstrated, the survival for the advanced laparoscopy is better compared to the basic laparoscopic group (*p* = 0.022). As expected, the operation technique has no influence on the survival curve, since reasons for dropout are not related to the technique itself, e.g., peritonitis, non-PD-related operations rendering in removal such as diverticulitis and failure of adequate dialysis because of thickening of the peritoneum.

## Discussion

After our RCT in 2017, we were disappointed about the functional outcome of PD catheter placement in the open and the laparoscopic group. After scrutinizing literature, we concluded that omental removal might improve our outcome, since omental wrapping causes most catheter failures. Also in our previous publication, we proved removal of the omentum to be successful; however, this is a challenging procedure [3]. Fixation of the omentum has been shown to be a safe and feasible alternative to prevent omental wrapping [9–11]. After several successful procedures, we confirmed these findings. Again checking our RCT, we concluded that the second most important reason for failure was migration of the catheter, and therefore, we started to use the abdominal wall fixation technique as described by others with success rates of 94 and 100% [7, 10]. These publications suggest that the advanced laparoscopic techniques lead to better outcome, but there are no randomized studies available. In the publications, there could be a selection bias based on improved experience or selection of patients for PD catheter placement. In our series, all consecutive patients were included, regardless of previous abdominal history, and therefore, this reflects a “real-world” PD patient population.

The outcome of both groups, basic versus advanced laparoscopy is similar, and there is no statically significant difference with a *p* = 0.1 in this small study. However, there is a trend toward better functional catheter outcome for advanced laparoscopic placement technique. We feel that the two described technical failures, one kinking of the subcutaneous part of the catheter and one catheter migration because of disconnection of the abdominal wall suture, are due to our learning curve and results can further improve with more experience. On the other hand, our learning curve should be taken into account explaining the possible better outcome of the advanced technique. Since the end of our RCT, we gained more experience. It is possible that the improved results can be explained by our increased experience and cannot be attributed to the new techniques.

To overcome the problems mentioned above, we will conduct a new multi-center RCT of basic laparoscopic placement versus advanced laparoscopic placement which includes fixation of the catheter and the omentum. We started including patients in our center in January 2020. However, because of the COVID-19 period, the inclusion has haltered and also the inclusion in other centers is problematic.

This study demonstrates another important point. A history of former placement of a PD catheter or a history of abdominal surgery, even with a midline laparotomy, seems no contraindication for recurrent PD catheter placement. The success rates of 88% and 80% are constantly with our former RCT and acceptable for a new attempt to have patients on peritoneal dialysis.

## Conclusion

This study demonstrated that there might be an advantage in functional outcome for placement of a laparoscopic peritoneal dialysis catheter with fixation of the omentum and catheter itself. It also demonstrates that there is an acceptable functional outcome for patients who need a redo-PD catheter or have abdominal surgery in the history. A new multi-center RCT will hopefully provide more definitive answers.

## Data Availability

Historical data already published are referred to. New data of feasibility study not in repository.
